# Effect of Seed Size on Pervaporation Performances Through FAU Zeolite Membrane

**DOI:** 10.3390/membranes15120355

**Published:** 2025-11-25

**Authors:** Alvin Rahmad Widyanto, Mikihiro Nomura

**Affiliations:** 1 Regional Environment Systems Course, Graduate School of Engineering and Science, Shibaura Institute of Technology, 3-7-5 Toyosu, Koto, Tokyo 135-8548, Japan; mc23501@shibaura-it.ac.jp; 2 Materials and Chemistry Program, College of Engineering, Shibaura Institute of Technology, 3-7-5 Toyosu, Koto, Tokyo 135-8548, Japan

**Keywords:** FAU zeolite membrane, seed size, secondary growth, pervaporation

## Abstract

Pervaporation is a compelling alternative to azeotrope-breaking and solvent dehydration due to lower energy demand and strong selectivity compared with distillation. FAU-type zeolite membranes combine large pore openings and hydrophilic frameworks with robust chemical stability, enabling water-selective separations from alcohols such as isopropanol and ethanol. Despite numerous synthesis routes, the role of seed crystal size in secondary growth, controlling nucleation density, intergrowth, and defect formation remains insufficiently quantified for FAU membranes under identical growth conditions. Here, FAU layers were fabricated on α-Al_2_O_3_ supports via secondary growth with varying seed sizes in the nanometer-to-micrometer range (72 nm to 6 μm). Zeolite crystal phase purity and morphology of membranes were assessed by XRD and SEM, with pervaporation of IPA/water 80 wt% at 75 °C quantified flux, separation factor, and permeance. We show that smaller seeds (95.51 nm) increase nucleation density, yielding thinner, more intergrown FAU layers with a higher separation factor but a modest trade-off in flux.

## 1. Introduction

The separation of isopropanol (IPA) from water represents a critical challenge in the chemical and biotechnology industries [1,2]. IPA, commonly produced through the fermentation of biomass, emerges from these processes at relatively low concentrations, typically below 5 wt%, making its efficient purification an economically and energetically demanding task [3]. Traditional distillation processes for IPA/water separation are notoriously energy-intensive, particularly given that the mixture forms an azeotrope at approximately 87.4 wt% IPA, which fundamentally limits the effectiveness of conventional thermal separation methods [4]. With increasing global emphasis on sustainable chemical production and energy efficiency, alternative separation technologies have garnered substantial interest.

Recent studies have demonstrated the potential of FAU zeolite membranes in various liquid separations [5,6,7]. For instance, researchers have successfully applied FAU membranes to the dehydration of isopropanol–water mixtures, achieving separation factors above 100 and permeate water concentrations exceeding 99 wt% [8,9]. The seeding strategy offers several advantages over direct in situ crystallization: it provides better control over membrane microstructure (including thickness, orientation, and crystal size distribution), minimizes the formation of intercrystalline defects that compromise separation capabilities, and improves reproducibility by decoupling nucleation from growth.

The size of seed crystals used in the seeding step has emerged as a crucial parameter influencing the final membrane quality and separation performance. Recent work on NaX zeolite membranes demonstrated that employing smaller seed particles of 750 nm, compared to larger micron-sized seeds, enhanced the membrane layer growth and successfully reduced defects due to the minimal gaps between seed particles [10]. Similarly, studies on FAU membrane synthesis using nano-sized seeds of approximately 50 nm reported exceptional gas separation performance [11]. For TS-1 zeolite membranes, systematic investigations revealed that medium-sized seed crystals were critical for achieving high catalytic performance, while seed size also influenced membrane thickness and morphology [12]. Research on silicalite-1 membranes further confirmed that seed crystal size affects membrane properties such as crystal orientation and separation efficiency [13].

While a handful of studies have explored seed size effects in pervaporation using other zeolite types, research specifically examining the effect of seed size on FAU zeolite membrane performance in pervaporation, particularly for IPA/water separation, remains surprisingly limited [6]. Moreover, the mechanistic understanding of how seed size governs membrane formation, including the interplay between seed layer packing density and crystal intergrowth during hydrothermal synthesis, has not been rigorously established for FAU-type zeolites in pervaporation. The present study was therefore designed to investigate the effect of seed crystal size, tuned via aging stirring and ball milling, on the pervaporation performance of FAU zeolite membranes for IPA/water separation. By varying seed particle size across a range from nano-scale to micron-scale dimensions, it aims to elucidate how seed size influences (1) the morphology, thickness, and defect characteristics of the resulting FAU membrane; (2) the pervaporation flux and separation performance of water/IPA; and (3) the underlying transport mechanisms governing permeation through the membranes.

## 2. Experimental Section

### 2.1. Materials

FAU zeolite powders and membranes were synthesized by using several materials including sodium silicate (Na_2_SiO_3_), sodium aluminate (NaAlO_2_), pure water, and sodium hydroxide (NaOH), which were purchased from Fujifilm Wako Pure Chemical Corporation (Tokyo, Japan). FAU zeolite membrane evaluation performances were used gas permeation and pervaporation test, which utilize hydrogen (H_2_), nitrogen (N_2_), and sulfur hexafluoride (SF_6_) gases, isopropyl alcohol (IPA, (CH_3_)_2_CHOH, Wako), ethanol (EtOH, C_2_H_6_O), methanol (MeOH, CH_3_OH, Wako), and pure water.

FAU-type zeolite powders and membranes were synthesized using sodium silicate (Na_2_SiO_3_), sodium aluminate (NaAlO_2_), sodium hydroxide (NaOH), and deionized water, all obtained from Fujifilm Wako Pure Chemical Corporation. The separation performance of the synthesized FAU membranes was evaluated by gas permeation and pervaporation tests. Hydrogen (H_2_) and sulfur hexafluoride (SF_6_) gases were used for gas permeation measurements, while isopropyl alcohol (IPA, (CH_3_)_2_CHOH, Wako) and deionized water were employed in pervaporation experiments.

### 2.2. FAU Zeolite Seed Preparation and Modification

FAU-type zeolite seed crystals were prepared following a modified procedure from a previous study [14], using synthetic molar composition of 12.8:1:17:675 (SiO_2_:Al_2_O_3_:Na_2_O:H_2_O), to investigate the influence of aging duration and mechanical treatment on particle size and crystallinity. The precursor solution was prepared by dissolving sodium silicate and sodium aluminate in deionized water, followed by the addition of sodium hydroxide in sodium aluminate solution. After achieving homogeneity, the two solutions were combined slowly and stirred for varying durations, specifically 4, 48, 144, and 168 h, to study the effect of aging time on seed development. Following the aging process, the mixture underwent hydrothermal treatment at 180 °C for 2 h to promote crystallization. The resulting FAU zeolite crystals were isolated via centrifugation, thoroughly washed to neutralize residual alkalinity, and dried.

To further reduce particle size, selected FAU zeolite seed samples, particularly those aged for 168 h, were subjected to ball milling using a pot mill rotator (PM 001, AS ONE, Osaka, Japan). Then, 2 g of FAU zeolite seed was milled at 200 rpm with 20 cm^3^ of 0.3 mm zirconia balls (AS ONE, Japan) in 40 cm^3^ of distilled water. The milling durations varied at 6, 10, 12, and 24 h to investigate the effect of mechanical treatment on seed morphology. After milling, the FAU seed suspension was separated from the zirconia balls using a sieve, and the collected sample was dried overnight at 100 °C.

### 2.3. FAU Zeolite Membranes Synthesis

FAU zeolite membranes were synthesized using a synthetic molar composition of SiO_2_:Al_2_O_3_:Na_2_O:H_2_O = 12.8:1:17:975 via a secondary growth method, with a focus on evaluating the impact of seed size on membrane formation [14]. Prior to seeding, 3 cm length of α-Al_2_O_3_ tubular supports (0.1 μm average pore diameter, 12 mm outer diameter, 1.5 mm thickness; Iwao Jiki Kogyo Co. Ltd., Seto, Aichi, Japan) were cleaned using ultrasonic treatment in water and acetone, followed by drying at 100 °C for 24 h. Using prepared FAU seed with different modifications, seeding was performed by immersing the supports three times in ethanol-based FAU seed suspensions (5 g L^−1^), with each immersion lasting 90 s and withdrawal at a rate of 0.5 cm s^−1^. The seeded supports were dried at 100 °C for 30 min after each dip to ensure adhesion of the seed layer. Hydrothermal crystallization was conducted at 90 °C for 24 h. FAU membranes were washed thoroughly with deionized water to remove residual synthesis solution and dried overnight at 100 °C.

### 2.4. Characterization and Evaluation

**Figure 1:**
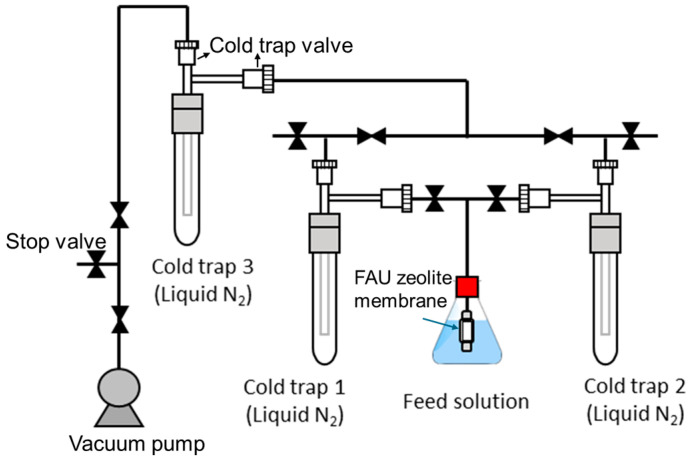
Schematic illustration of PV apparatus setup.

The structural and morphological properties of FAU zeolite seeds and membranes were characterized using X-ray diffraction (XRD) and field emission scanning electron microscopy (FESEM). XRD analysis (Rigaku Ultima IV, Tokyo, Japan, Cu Kα radiation, λ = 1.5406 Å) was performed over a 2θ range of 5° to 40° to confirm phase purity and crystallinity. Peak intensity and broadening were used to assess the effects of aging and ball milling on seed structure, with particular attention to the (111) reflection near 6.2°, indicative of FAU-type framework integrity. FESEM imaging (JEOL/JSM-7610F, Tokyo, Japan) provided detailed surface and cross-sectional views of both seed particles and membrane layers. These images were used to evaluate crystal intergrowth, membrane thickness, and fragmentation resulting from mechanical treatment. Elemental distributions were observed via energy-dispersive X-ray spectroscopy (EDX), offering insights into FAU seed penetration on support. Dynamic light scattering (DLS) was employed to measure the hydrodynamic particle size distribution of FAU seed suspensions using BECKMAN COULTER/DelsaMax PRO (Beckman Coulter Inc., Brea, CA, USA), complementing SEM observations and revealing behavior of dispersion in liquid media. Filter paper was used to separate the suspended powder and solution. Crystallite sizes were estimated from XRD peak broadening using the Williamson–Hall method [15]. Gas permeation tests were conducted using single gases (H_2_, N_2_, SF_6_) at room temperature to evaluate membrane selectivity and defect density. Feed pressure was maintained at ~0.4 MPa, and permeate flow rates were measured using bubble flow and pressure difference techniques depending on the permeance recorded [16]. Pervaporation performance was assessed using an 80 wt% alcohol/water mixture at 75 °C. Flux and separation factors were calculated to quantify the trade-off between transport and selectivity. Membranes synthesized from seeds with varying aging and ball milling durations were compared to elucidate the influence of seed size on membrane structure and separation efficiency. As illustrated in Figure 1, the setup consists of a stainless-steel membrane cell connected to a feed tank, preheater, and vacuum system, with permeate collected under vacuum (<0.1 kPa) and condensed in a cold trap. Flux (J), separation factor (SF), and permeance (P) were obtained from Equations (1)–(3), respectively.

(1) $$J=\frac{m}{A\times t}$$

where *m* is the mass of the permeate (kg), *A* is the effective membrane area (m^2^), and *t* is the time (h).

(2) $$\alpha_{i/j}=\frac{\left(y_{i}/y_{j}\right)}{\left(x_{i}/x_{j}\right)}$$

where *y* and *x* represent the weight fractions of components *i* and *j* in permeate and feed, respectively.

(3) $$P_{i}=\frac{J_{m,i}}{\left(p_{f,i}-p_{p,i}\right)}$$

where *J_m_*_,*i*_ is the molar flux of component *i*, and *p_f_*_,*i*_ and *p_p_*_,*i*_ are its partial pressures in feed and permeate.

## 3. Results and Discussion

### 3.1. FAU Zeolite Seed Characterizations

**Figure 2:**
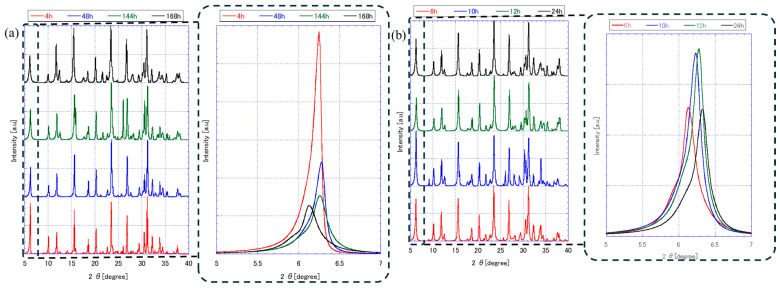
XRD characterization of FAU zeolite seed prepared by different (**a**) aging stirring and (**b**) ball milling times with zoom in diffraction on 6°.

Figure 2 presents the XRD patterns of FAU zeolite seeds prepared under varying stirring and ball milling durations. The diffraction peaks observed across all seeds confirm the presence of an FAU-type crystalline structure, with characteristic reflections at 2θ values corresponding to ~6.23° (111), 10.11° (220), 11.86° (311) 15.58° (331), 18.58° (333), 20.25° (440), 22.66° (620), 23.485° (533), 26.89° (642), 29.42° (733), 30.55° (822), 31.20° (555), 32.24° (840), 32.98° (753), and 33.86° (664) planes. It was matched with JCPDS No. 39-1380 standard [17,18]. Notably, the intensity and sharpness of these peaks vary depending on the seed preparation conditions.

The effect of aging stirring time on FAU zeolite seed formation is a critical factor influencing the crystallinity and structural character of FAU zeolites. Based on Figure 2a, aging times of 4h, 48h, 144h, and 168h show distinct differences in peak sharpness and intensity, which reflect the degree of crystallinity and phase development. At shorter aging times (4 h), the XRD peaks are sharper and more intense, particularly at the 111 plane around 6.23° 2θ. This suggests that the FAU framework forms more robustly under these conditions, likely due to a higher concentration of reactive species and less structural rearrangement [19].

As the aging time increases to 48h and beyond, the XRD peaks begin to broaden and decrease in intensity. This trend indicates a reduction in crystallinity, possibly due to the formation of more siliceous species in the liquid phase. These species can interfere with the orderly arrangement of aluminosilicate frameworks, leading to less defined FAU phase precursors [20,21]. By 168h, the disruption in the FAU framework becomes more pronounced, as evidenced by the diminished intensity of the 111 reflections. This suggests that prolonged aging may lead to partial amorphization with smaller crystals size forming.

FAU seed produced from 168h aging were subjected to different ball milling durations, 6 h, 10 h, 12 h, and 24 h, respectively (Figure 2b). All samples retain FAU-type peaks, confirming structural identity; however, peak intensity and sharpness decrease with longer milling times. Seeds milled for 6 h exhibit sharp, intense peaks, indicating high crystallinity and larger crystallite size. In contrast, 24 h milling produces broad, weak peaks, suggesting significant amorphization and lattice strain [22]. The zoomed-in view highlights progressive peak broadening and intensity loss, consistent with mechanical fragmentation and reduced structural order. Interestingly, the (111) peak intensity for 10 h and 12 h milling is slightly higher than that for 6 h. This can be attributed to partial fragmentation combined with preserved crystallinity. At these intermediate milling durations, the seeds are sufficiently broken down to reduce agglomeration and improve packing during XRD measurement, while maintaining enough structural integrity to produce strong diffraction. This phenomenon is consistent with findings by Mazzeo et al. [23], who demonstrated that mechanochemical treatments initially enhance diffraction quality due to improved particle dispersion and reduced agglomeration before extensive comminution and defect formation occur.

As shown in Table 1, seeds aged for 4 h display moderate crystallinity (83.55%), which increases to a maximum of 95.53% at 48 h, indicating that intermediate aging promotes well-ordered FAU frameworks. However, further aging to 144–168 h reduces crystallinity to ~80–84%, likely due to structural disorder and partial amorphization caused by prolonged exposure to siliceous species in the precursor solution. This trend suggests that excessive aging disrupts the aluminosilicate network, leading to smaller, less crystalline particles [24,25]. Ball milling introduces a different effect: short milling (6 h) enhances crystallinity to 91.44%, possibly due to improved dispersion and reduced agglomeration. In contrast, extended milling (10–24 h) decreases crystallinity to ~79–88%, reflecting lattice strain and amorphization from mechanical stress [26]. Micro strain values obtained from the Size–Strain Plot (SSP) method provide insight into lattice distortion within FAU seeds under different preparation conditions [27,28]. During aging, micro strain remained relatively low (3.94–5.40%), indicating that extended aging primarily affected particle size and crystallinity rather than introducing significant internal stress. In contrast, ball milling caused a marked increase in micro strain, reaching 6.86% at 6 h and peaking at 8.28% at 10 h, before slightly decreasing at 24 h (6.03%). This trend suggests that mechanical treatment introduces lattice strain through fragmentation and defect formation, while prolonged milling leads to partial amorphization [22], reducing measurable strain.

**Figure 3:**
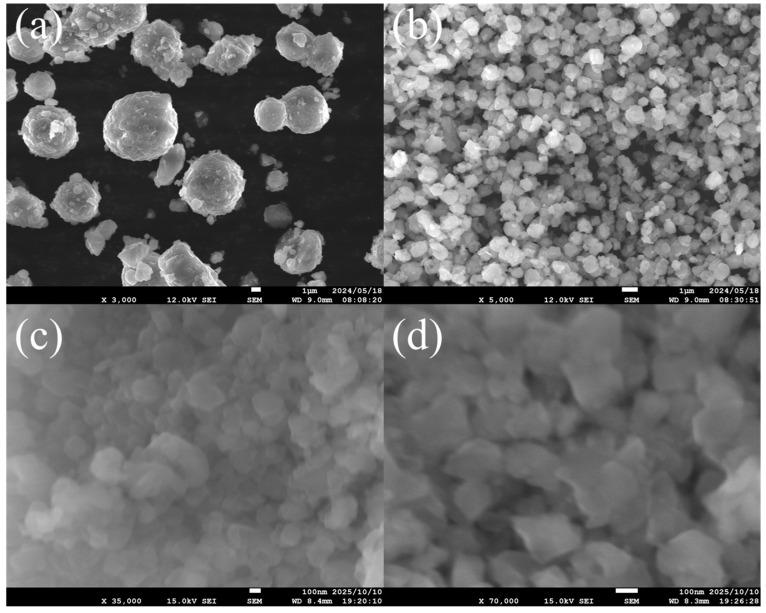
SEM characterization of FAU zeolite seed prepared at various aging stirring times at (**a**) 4 h, (**b**) 48 h, (**c**) 144 h, and (**d**) 168 h.

Figure 3 illustrates SEM images of FAU zeolite seeds prepared under different aging durations (4 h, 48 h, 144 h, and 168 h). The micrographs reveal a clear morphological evolution as aging stirring time increases. At 4 h, the seeds appear as large, aggregated particles with relatively smooth surfaces, indicating rapid nucleation (2h at 180 °C) and growth under high supersaturation conditions [29]. By 48 h, the FAU seed particles become smaller and more uniformly dispersed, suggesting a shift toward slower growth kinetics and increased nucleation density. At 144 h and 168 h, the seeds exhibit highly fragmented and irregular morphologies, consistent with partial amorphization and structural disorder. These prolonged aging conditions promote the breakdown of larger crystals into smaller fragments [19].

**Figure 4:**
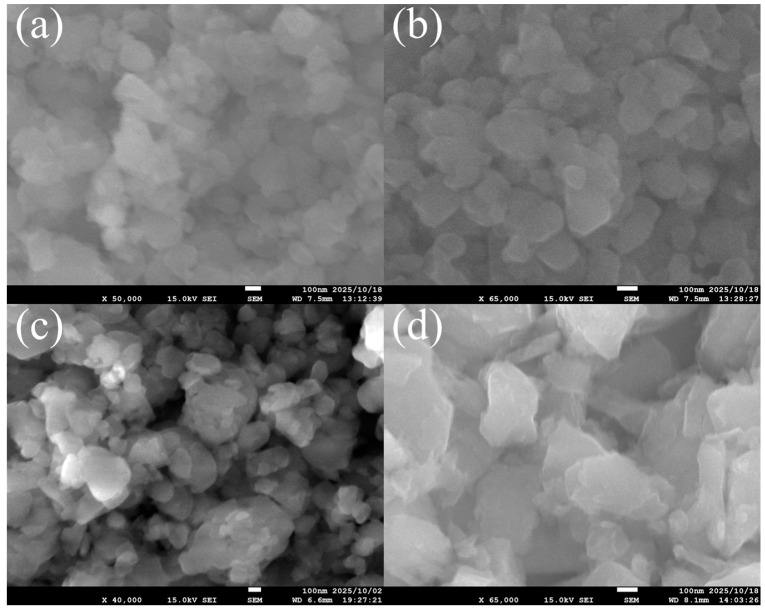
SEM characterization of FAU zeolite seed prepared various ball milling times at (**a**) 6 h, (**b**) 10 h, (**c**) 12 h, and (**d**) 24 h.

Figure 4 presents SEM images of FAU zeolite seeds after ball milling for 6 h, 10 h, 12 h, and 24 h. At 6 h, the seeds remain relatively large and well-defined, with smooth surfaces. This indicates that short milling preserves structural integrity and crystallinity, which is advantageous for forming dense membranes with fewer defects, although the lower nucleation density may lead to thicker layers. At 10 h and 12 h, the particles are smaller and more fragmented, and surface irregularities begin to appear. These intermediate milling durations enhance nucleation density during secondary growth, promoting thinner and more intergrown layers. By 24 h, the seeds exhibit severe fragmentation and the smallest particle sizes among the samples. Prolonged milling introduces significant amorphization and lattice strain, confirmed by diminished XRD peak intensity. This trend is consistent with studies on ZSM-5 and other zeolites, which show that extended milling reduces micropore volume and crystallinity due to defect formation and framework collapse [30,31].

Table 1 summarizes the crystallite size (from XRD) and particle size (from SEM and DLS) analysis of FAU zeolite seeds prepared under different aging and ball milling conditions. These data provide a quantitative link between synthesis parameters and the structural characteristics observed in Figure 2, Figure 3 and Figure 4.

As aging time increases from 4 h to 168 h, both crystallite size and particle size decrease significantly. For example, seeds aged for 4 h exhibit a crystallite size of 36.7 ± 3.7 nm and a particle size of 6106.99 ± 1075.92 nm, whereas those aged for 168 h show a crystallite size of 25.7 ± 1.2 nm and a particle size of only 125.04 ± 23.21 nm. This trend aligns with the XRD patterns in Figure 2a, were prolonged aging leads to peak broadening and reduced intensity, indicating lower crystallinity. SEM images in Figure 3 confirm this observation: short aging produces large, aggregated crystals with smooth surfaces, while extended aging results in fragmented, irregular particles.

Ball milling further refines particle size without significantly altering crystallite size. Mukhtar et al. [32] studied the effect of ball milling on commercial synthetic zeolite; the particle size was reduced from >45 μm to ~200–300 nm. Seeds aged 168 h and milled for 6 h show a particle size of 109.80 ± 4.04 nm, while 24 h milling reduces this to 72.08 ± 6.54 nm. However, XRD data in Figure 2b reveal that prolonged milling introduces lattice strain and amorphization, as evidenced by peak broadening and intensity loss. SEM images in Figure 4 corroborate this: short milling preserves structural integrity, whereas extended milling produces highly fragmented particles with rough surfaces.

Comparing SEM and DLS data provides additional insight into particle dispersion. SEM measures dry-state aggregates, often showing large clusters (>6 μm for 4 h aging), while DLS reflects hydrodynamic size in suspension, which is much smaller (~432 nm for the same sample). This indicates that dispersion partially breaks down aggregates but does not fully separate primary crystallites. Xue et al. [33] demonstrated that employing water as a dispersant can result in the smallest particle size of nanoparticles compared with organic and aqueous dispersant for DLS test. For long aging and milling, SEM shows fragmented particles (~72–125 nm), and DLS confirms smaller hydrodynamic sizes (~297–372 nm), though still larger than the crystallite sizes from XRD (~26–28 nm). The gap between XRD and DLS values suggests residual agglomeration even after milling [34]. Polydispersity further supports this: low polydispersity for aged samples without milling indicates uniformity, while high polydispersity for long milling times (e.g., 13.00 at 24 h) reflects heterogeneous fragmentation and possible amorphization.

### 3.2. FAU Zeolite Membrane Characterizations

**Figure 5:**
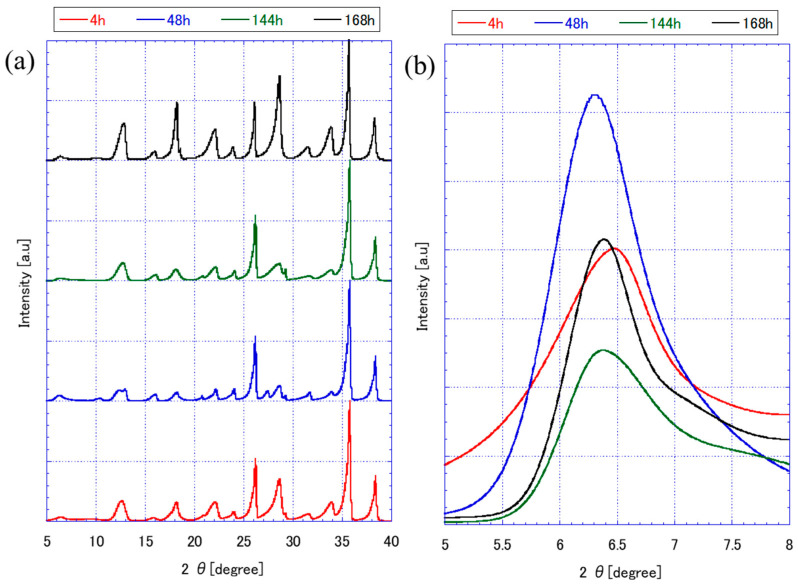
XRD characterization of FAU zeolite membrane prepared from seed with different aging stirring times on (**a**) full range and (**b**) zoom in diffraction on 6°.

Figure 5 shows XRD patterns of FAU zeolite membranes synthesized using seeds aged for 4 h, 48 h, 144 h, and 168 h. All membranes exhibit characteristic FAU peaks at 6.32, 10.48, 16.08, 18.19, 20.68, 22.23, 24.01, 29.24, 31.68, and 33.95°, confirming the successful formation of the FAU framework regardless of aging time. However, it was also observed that NaP peaks at 12.96, 22.27, and 28.64°. Other peaks from α-Al_2_O_3_ were observed at 26.11, 35.69, and 38.31°, respectively. The coexistence of FAU and NaP phases suggests that the synthesis conditions favored partial transformation or competitive crystallization during secondary growth [11]. NaP formation is typically associated with silica-rich environments or prolonged aging, where the chemistry of the precursor solution shifts toward conditions that stabilize alternative aluminosilicate frameworks [35]. The differences in XRD patterns are not a direct effect of aging time but rather a consequence of seed morphology and size, which aging controls. Larger, well-crystallized seeds from short aging (4 h) promote dense FAU layers with minimal secondary phases, as reflected by sharp FAU peaks and negligible NaP signals. Membranes derived from 48 h aged seeds display the most intense (111) and well-defined peaks. In contrast, smaller seeds from prolonged aging (144–168 h) increase nucleation density but also introduce structural heterogeneity, creating conditions where NaP can form alongside FAU. Similar trends were reported for NaX membranes, where smaller seeds improved intergrowth and reduced defects, while larger seeds created intercrystalline gaps and allowed impurity phases to develop [10].

**Figure 6:**
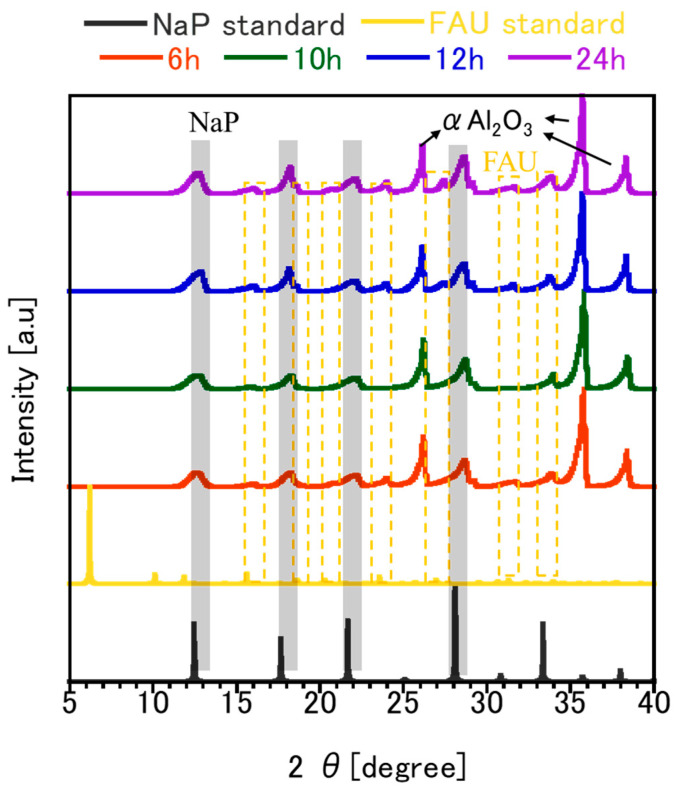
XRD characterization of FAU zeolite membrane prepared from seed with different ball milling times; dashed line denotes FAU peaks; black transparent line denoted NaP peaks, while arrow represent support peaks.

Figure 6 shows XRD patterns of FAU zeolite membranes synthesized using seeds subjected to different ball milling durations (6 h, 10 h, 12 h, and 24 h), alongside FAU and NaP standards for comparison. All membranes exhibit small characteristic FAU peaks (16.00, 18.25, 20.80, and 23.98°), NaP peaks (12.63, 22.27, and 28.66°), and α-Al_2_O_3_ peaks (26.21, 35.78, and 38.39°), respectively. However, the intensity and sharpness of these peaks vary significantly, reflecting changes in crystallinity and structural integrity caused by mechanical treatment. Prolonged milling introduces defects such as dislocations and micro strain, which disrupt long-range order and reduce crystallinity. The presence of NaP as an impurity phase in FAU membranes introduces a complex interplay between adsorption-driven selectivity and defect-mediated transport. NaP possesses smaller pore openings (~0.36 nm) and a more one-dimensional channel system compared to FAU’s three-dimensional network (~0.74 nm), which significantly alters its transport characteristics. Its strong hydrophilicity can locally enhance water adsorption, potentially contributing to high water selectivity in membranes where NaP domains coexist with FAU crystals [36]. However, NaP formation is often associated with structural heterogeneity and grain-boundary defects, which create non-zeolitic pathways that facilitate IPA permeation and reduce overall permselectivity [11,35].

**Figure 7:**
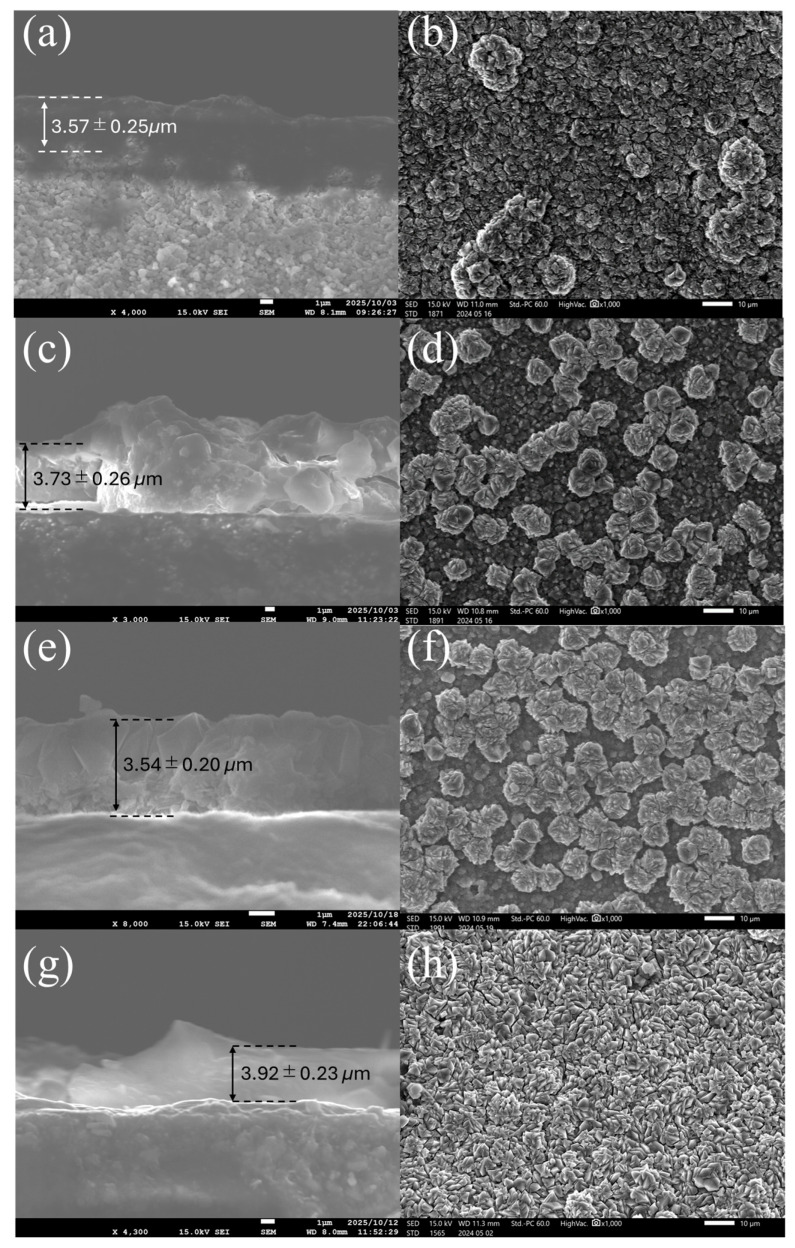
Cross-section and surface SEM characterization of FAU zeolite membrane prepared from FAU seed prepared from various aging stirring times at (**a**,**b**) 4 h, (**c**,**d**) 48 h, (**e**,**f**) 144 h, and (**g**,**h**) 168 h.

Figure 7 shows cross-sectional and surface SEM images of FAU zeolite membranes synthesized using seeds aged for 4 h, 48 h, 144 h, and 168 h. The measured thicknesses, 3.57 ± 0.25, 3.73 ± 0.26, 3.54 ± 0.20, and 3.92 ± 0.23 μm, respectively, indicate that aging duration does not significantly affect membrane thickness under the applied synthesis conditions. However, the surface morphology varies considerably, which strongly influences membrane performance. Membranes prepared from 4 h aged seeds exhibit dense, compact layers with smooth intergrowth and relatively large, well-faceted crystals. This morphology reflects the high crystallinity and larger particle size of seeds formed under short aging conditions, which typically minimizes grain-boundary defects and enhances separation performance, albeit at the expense of flux due to the thicker layer [21]. Similar observations have been reported for FAU membranes synthesized under controlled growth conditions, where larger seeds promote robust intergrowth but limit transport properties [37]. At 48 h aging, the surface becomes more uniform and finely grained, suggesting increased nucleation density from smaller seed particles. Membranes derived from 144 h aged seeds display a more fragmented surface with irregular crystal packing. Although thickness remains comparable, the rougher surface indicates reduced crystallinity and smaller seed size, which can enhance flux but introduce grain-boundary defects that compromise separation performance. Prolonged aging is known to produce siliceous species and structural disorder, leading to such morphological changes [38]. FAU membranes synthesized using 168 h aged seeds show the most granular and densely packed surface. The increased fragmentation of seed particles likely boosts nucleation density, but the risk of intercrystalline voids and secondary phase formation (e.g., NaP) becomes higher under these conditions, as observed in FAU systems.

**Figure 8:**
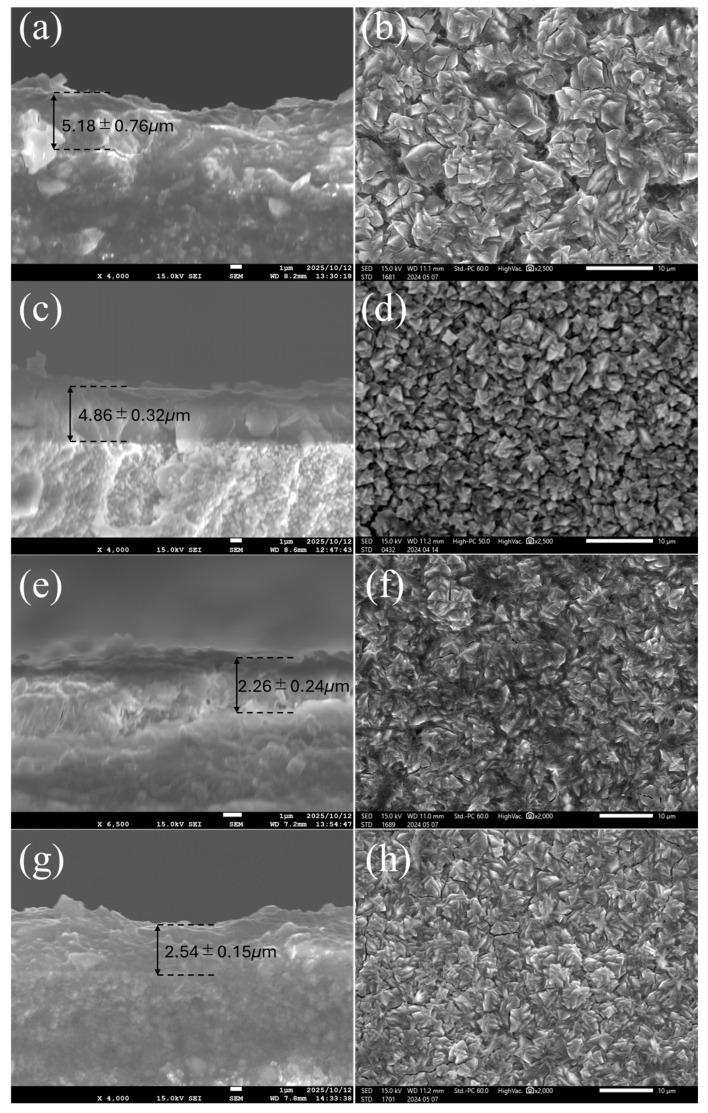
Cross-section and surface SEM characterization of FAU zeolite membrane prepared from FAU seed at various ball milling times: (**a**,**b**) 6 h, (**c**,**d**) 10 h, (**e**,**f**) 12 h, and (**g**,**h**) 24 h.

Figure 8 presents cross-sectional and surface SEM images of FAU zeolite membranes synthesized using seeds subjected to ball milling for 6 h, 10 h, 12 h, and 24 h. The measured thicknesses, 5.18 ± 0.76, 4.86 ± 0.32, 2.26 ± 0.24, and 2.54 ± 0.15 μm, respectively, show a clear trend: prolonged milling significantly reduces membrane thickness. This reduction is attributed to the fragmentation of seed particles, which increases nucleation density during secondary growth and accelerates lateral intergrowth, resulting in thinner layers. Membranes prepared from seeds milled for 6 h exhibit relatively thick and compact layers with smooth, well-intergrown surfaces. The large seed fragments preserve crystallinity, enabling robust secondary growth and minimizing grain-boundary defects. Such morphology typically favors high separation performance, although the increased thickness may limit flux. At 10 h milling, the membrane thickness decreases slightly, and the surface becomes more uniform and finely grained. This suggests that moderate milling enhances nucleation density without severely compromising crystallinity, producing thinner membranes with improved intergrowth. Similar observations have been reported for NaA membranes, where intermediate seed sizes achieved by controlled milling optimize coverage and reduce defect formation [39]. Membranes derived from 12 and 24 h milled seeds show a pronounced reduction in thickness and increased surface roughness. The cross-sectional view reveals a less compact structure, and the surface appears more fragmented. These features reflect the onset of structural disorder and lattice strain caused by extended milling, which can enhance flux but introduce grain-boundary defects that compromise separation performance. The strong correlation between milling time and thickness compared to aging is explained by the magnitude of size reduction: ball milling produces much smaller seeds than aging, dramatically increasing nucleation density and accelerating lateral growth, which closes the membrane layer earlier and limits vertical growth. In contrast, aging only moderately reduces seed size, so its effect on nucleation density, and thus thickness, is weaker. This mechanism aligns with previous reports that smaller seeds promote rapid surface coverage and thin, continuous layers [11], while excessive fragmentation can lead to non-selective pathways.

**Figure 9:**
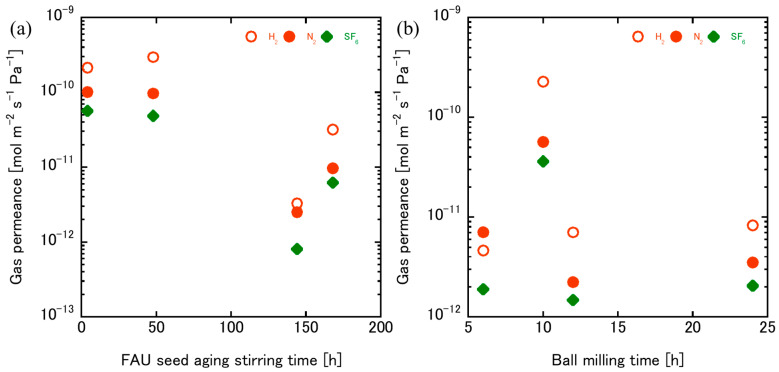
Gas permeation of H_2_, N_2_, and SF_6_ on FAU zeolite membrane using FAU seed prepared by different (**a**) aging stirring and (**b**) ball milling times.

Although the kinetic diameters of H_2_ (0.289 nm), N_2_ (0.364 nm), and SF_6_ (0.55 nm) are smaller than the FAU pore size (~0.74 nm), these gases are not used to evaluate molecular sieving but to detect non-zeolitic pathways such as intercrystalline gaps and grain-boundary defects. In an ideal FAU membrane with perfect intergrowth, all three gases should permeate easily, and selectivity would follow Knudsen diffusion ratios. However, when structural defects exist, SF_6_ permeance increases disproportionately because its larger size makes it more sensitive to non-zeolitic voids. Therefore, SF_6_ permeance serves as a defect indicator: higher SF_6_ permeance suggests incomplete coverage and intercrystalline gaps, while lower values indicate dense, defect-minimized layers. The H_2_/SF_6_ selectivity ratio is often used as a quantitative measure of membrane integrity, with higher ratios corresponding to better structural quality. The gas permeation results in Figure 9 provide a clear picture of how FAU seed preparation, through aging and ball milling, affects membrane structure and separation properties. For aging stirring (Figure 9a), membranes prepared with 4 h and 48 h aging exhibited high SF_6_ permeance (~10^−10^ mol·m^−2^·s^−1^·Pa^−1^), indicating the presence of intercrystalline gaps. Similar trends have been reported for FAU and NaX membranes, where insufficient aging leads to non-selective transport pathways [35]. In contrast, prolonged aging (144–168 h) produced smaller, more uniform seeds that enhanced nucleation density and crystal packing, reducing SF_6_ permeance to ~10^−11^–10^−12^ and confirming the formation of dense FAU layers with minimal grain-boundary defects. For ball milling (Figure 9b), the effect is even more pronounced: milling generates much smaller particles that increase nucleation density and penetrate deeper into the support, forming highly compact layers with SF_6_ permeance near 10^−12^. Membranes prepared from seeds milled for 6 h exhibit the lowest permeance for all gases, indicating dense, defect-minimized layers. The observation that N_2_ permeance was higher than H_2_ for the membrane prepared from seeds milled for 6 h is unusual because, under ideal conditions, permeance should follow Knudsen diffusion, where smaller molecules (H_2_) permeate faster than larger ones (N_2_). Although H_2_ has a smaller kinetic diameter (0.289 nm) than N_2_ (0.364 nm), its very low polarizability and weak interaction with the FAU framework result in minimal adsorption, limiting its overall transport. In contrast, N_2_ exhibits stronger van der Waals interactions with the aluminosilicate surface, which enhances adsorption and compensates for its slightly larger size [40]. This effect becomes more pronounced in dense, defect-minimized membranes formed from short milling durations, where transport is dominated by surface interactions within micropores rather than through non-selective gaps. Consequently, N_2_ permeance can surpass H_2_ despite the size difference, reflecting the combined influence of adsorption affinity and diffusion pathways in FAU zeolite membranes. Short milling preserves seed crystallinity while reducing particle size enough to improve surface coverage. However, as milling time increases to 10–12 h, permeance rises significantly, reflecting structural fragmentation and reduced crystallinity. Extended milling to 24 h slightly lowers permeance compared to 12 h but remains higher than at 6 h, suggesting that excessive milling introduces amorphization and lattice strain, which disrupt micropore networks and compromise selectivity.

### 3.3. FAU Zeolite Membrane Pervaporation Evaluation

**Figure 10:**
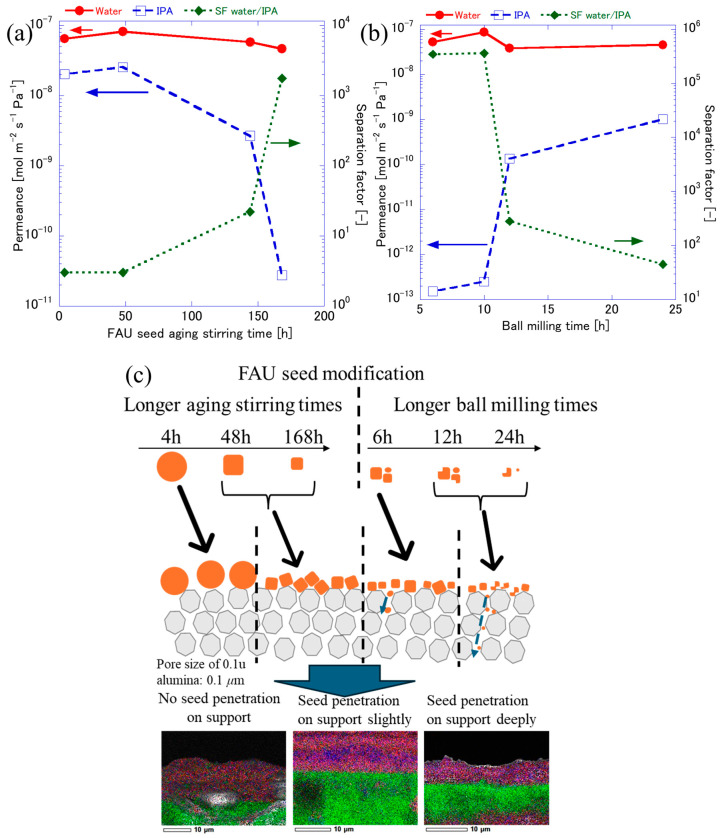
PV results of 80 wt% IPA on FAU zeolite membrane using FAU seed prepared by different (**a**) aging stirring and (**b**) ball milling times; (**c**) schematic illustration of influence of FAU seed size on PV performance.

Figure 10 demonstrates how FAU seed modification, through aging stirring and ball milling, affects the pervaporation performance of membranes separating 80 wt% IPA/water mixtures at 75 °C. The results reveal a strong dependence of separation performances on seed preparation, while water permeance remains relatively stable across all conditions. As shown in Figure 10a, water permeance stays nearly constant at ~10^−7^ mol·m^−2^·s^−1^·Pa^−1^, regardless of aging duration, indicating that flux is not significantly influenced by seed size in this range. In contrast, IPA permeance decreases markedly from ~10^−8^ at 4 h to ~10^−10^ at 168 h, leading to a dramatic increase in the separation factor from ~3.20 to over 1759. This improvement in the separation factor can be attributed to the formation of smaller, more uniform seeds during prolonged aging, which enhances nucleation density and promotes dense FAU layers with fewer intercrystalline gaps. This behavior is attributed to the adsorption–diffusion mechanism governing transport in FAU zeolite membranes. Water molecules strongly adsorb onto the hydrophilic FAU framework and diffuse through its micropores [41]; thus, water permeance is primarily controlled by intrinsic pore properties and remains stable even as membrane thickness increases. In contrast, IPA molecules exhibit weaker adsorption and rely more on non-zeolitic pathways such as intercrystalline gaps. When smaller seeds or thicker membranes reduce defect density and eliminate these gaps, IPA transport is significantly suppressed, resulting in a dramatic drop in IPA permeance and a corresponding rise in separation factors. Sawamura et al. [8] demonstrated that FAU membranes achieved stable water permeance with high water permselectivity (~10^3^) for 20/80 IPA–water mixtures, attributed to dense intergrowth and minimized non-zeolitic pores. In addition, Matsukata et al. [42] showed that optimizing seed size via seeding conditions yields compact FAU layers, suppressing IPA diffusion. Figure 10b shows that short milling (6 and 10 h) yields extremely low IPA permeance (~10^−13^) and very high separation factor (>10^5^), reflecting dense, defect-minimized membranes formed from relatively intact seeds. Wang et al. [6] demonstrated that FAU membranes synthesized from 50 nm seeds achieved high separation factors (>500) with stable flux, owing to the dense packing and minimal intercrystalline voids provided by nano-scale particles. Although, all membranes prepared by ball milled seed modification generated NaP peaks, different milling times contributed different separation properties. Fasano et al. [43] elucidated the contribution of nanopore hydrophilicity and surface barriers on the overall water transport through zeolite crystals. Pore blockages or narrowing caused by moderate milling penetrate the support more effectively, increasing nucleation density and enabling dense membrane formation that significantly limit IPA permeance (<10^12^) (Figure 10c). However, as milling time increases to 24 h, IPA permeance rises to ~10^−10^ and separation factor drops to ~10^2^, despite water permeance remaining constant. This suggests that IPA permeance is dominated by non-selective pathways (e.g., intercrystalline gaps and grain-boundary defects). This decline in the separation factor is linked to structural disorder and amorphization caused by excessive milling, which introduces non-uniform crystal packing and the presence of secondary phases or amorphous regions within the membrane layer.

**Figure 11:**
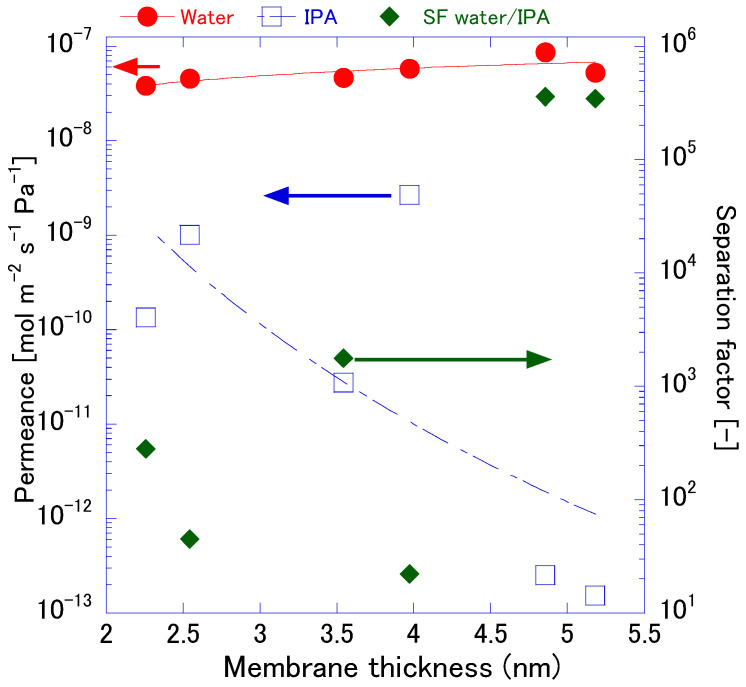
Correlation membrane thickness and PV performances.

The relationship between membrane thickness, water, and IPA permeance and the resulting separation factor for FAU zeolite membranes is shown in Figure 11. Interestingly, water permeance remains relatively stable across the thickness range (~10^−7^ mol·m^−2^·s^−1^·Pa^−1^). In contrast, IPA permeance shows a strong dependence on thickness. At around 2.5 µm, IPA permeance is close to ~10^−9^ mol·m^−2^·s^−1^·Pa^−1^, but it decreases sharply to nearly $10^{-13}$ as thickness approaches 5 µm. Thicker membranes effectively suppress IPA transport, likely due to the reduced defect density and longer diffusion paths. This trend reflects the dual effect of reduced defect density and extended diffusion paths on thicker layers, which collectively enhance the separation factor. Sawamura et al. [8] reported similar behavior for FAU membranes in IPA/water vapor permeation, where compact layers prepared via two-step crystallization exhibited high water permselectivity (α > 1000) and minimized non-zeolitic pores, thereby limiting IPA transport. In contrast, Villaluenga et al. [44] observed that for polymeric membranes, flux decreased proportionally with thickness, but the separation factor remained nearly constant beyond a critical thickness, indicating that separation was governed by intrinsic sorption and diffusion properties rather than structural imperfections. These differences underscore the unique role of microstructural integrity in inorganic membranes: while polymeric systems primarily exhibit a flux–thickness trade-off, FAU zeolite membranes leverage thickness to mitigate grain-boundary defects, achieving a sharp rise in the separation factor.

**Figure 12:**
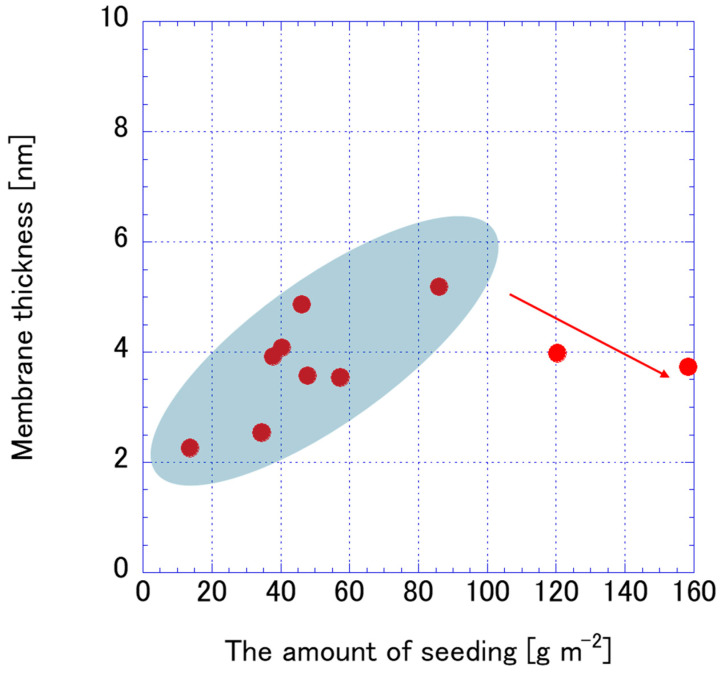
Correlation of amount of seeding and membrane thickness.

The relationship between the amount of FAU zeolite seeds applied during secondary growth and the resulting membrane thickness is subtle but important for optimizing membrane performance. As shown in Figure 12, increasing the seeding amount generally promotes better surface coverage, which accelerates nucleation and lateral crystal growth. Membranes prepared from seeds aged for 4–168 h maintain relatively constant thickness (3.54–4.08 μm) despite large variations in seeding amount (37.78–158.44 g·m^−2^). This weak correlation suggests that seed size and dispersion quality, rather than nominal seeding quantity, dominate membrane growth. In contrast, ball milling significantly reduces thickness, with membranes from 12 to 24 h milled seeds reaching 2.26–2.54 μm, even at low seeding amounts (13.64–34.55 g·m^−2^). This agrees with findings by Wang et al. [11], who achieved 2.7 μm thick FAU membranes using nano-sized seeds (~50 nm). Similarly, Matsukata et al. [42] observed that optimizing seed size and slurry conditions was critical for forming crack-free FAU membranes, while excessive fragmentation introduced structural heterogeneity. To the best of our knowledge, the large amount of seeding was prone to peel off; thus, membrane thickness formation did not correlate with the number of seeding at specific points.

**Figure 13:**
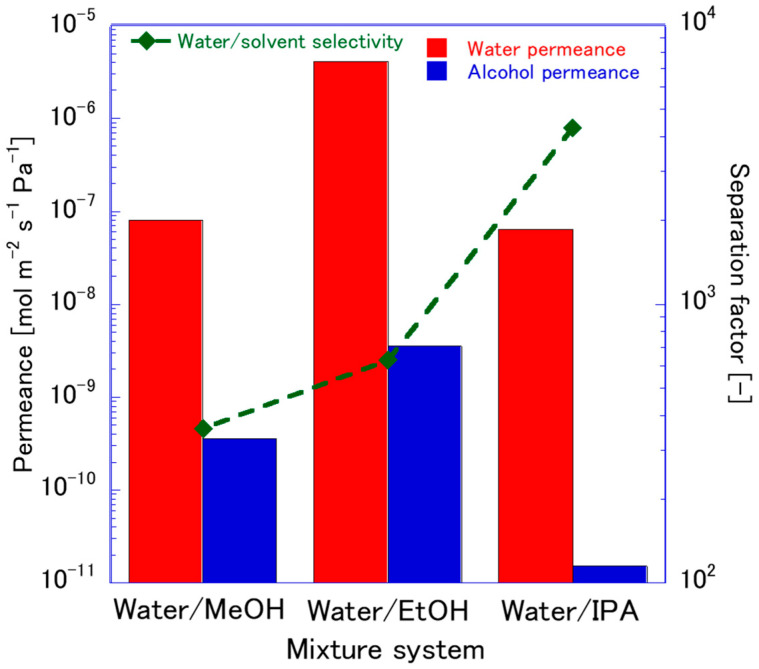
PV performance of FAU zeolite membrane synthesized using seed crystals aged 168 h and ball milled for 10 h, tested with various organic solvents.

Figure 13 illustrates the pervaporation performance of the FAU membrane, which was fabricated using FAU seeds aged for 168 h and ball milled for 10 h under the same conditions (80 wt% of alcohol/water mixtures at 75 °C). This seed treatment produced small, moderate crystalline particles that enhanced nucleation density during secondary growth, resulting in a thin membrane layer with high flux. Across different organic solvents, it consistently exhibited high water flux, attributed to the hydrophilic nature and large pore openings of the FAU framework. Performance variations across different solvents are not only controlled by adsorption–diffusion but also by solvent polarity, molecular size, and membrane microstructure [6]. For instance, highly polar solvents (e.g., MeOH) compete more strongly with water for adsorption sites in FAU pores, significantly reducing water selectivity and separation factors, whereas fewer polar solvents (e.g., IPA) result in higher selectivity [45,46]. Wang et al. [6] further demonstrated that FAU pervaporation performance correlates with the polarity index of the permeate, reinforcing the role of adsorption affinity in controlling flux. The framework’s large pore diameter (~0.74 nm) allows water molecules to diffuse readily, while larger or more weakly adsorbing organics are sterically and energetically hindered, amplifying the polarity-driven separation. However, structural factors such as layer thickness, intergrowth quality, and presence of secondary phases (e.g., NaP) modulate this intrinsic effect. Thin, well-intergrown membranes, typical of moderate milling, enhance separation performance by reducing intercrystalline defects and promoting dense FAU membrane formation.

**Figure 14:**
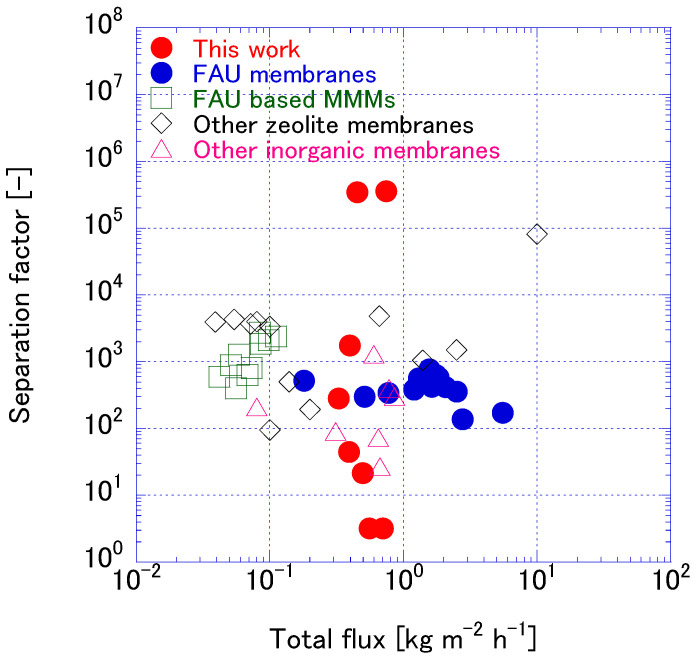
Comparison of PV performances with literature.

Figure 14 compares the PV performance of the FAU zeolite membrane developed in this study with various membranes reported in the literature, including other FAU, FAU-based mixed-matrix, zeolite, and inorganic membranes (Table S1). The comparison focuses on total flux and separation factors under similar test conditions for IPA/water mixtures. The FAU membrane synthesized using seed crystals aged for 168 h and ball milled for 6 and 10 h demonstrates superior performance in terms of water flux and competitive separation capabilities. The well-intergrown zeolite layer and the hydrophilic nature of the FAU framework facilitates water transport.

## 4. Conclusions

The study demonstrated that FAU zeolite seed size, controlled via aging stirring and ball milling, plays a pivotal role in determining the morphology, crystallinity, and performance of FAU membranes synthesized by secondary growth. Smaller seed particles, obtained through prolonged aging (up to 168 h) and moderate ball milling (10 h), significantly enhance nucleation density, leading to thinner membrane layers with higher flux during pervaporation. However, this comes at the cost of reduced crystallinity and increased grain-boundary defects, which can compromise separation factors. The performance of membranes, synthesized using seeds aged for 168 h and ball milled for 6 and 10 h, demonstrated attractive performances. It achieves modest flux and high separation factors values, positioning it favorably against membranes reported in the literature. Recently, the use of nano-sized seed provided advantages to obtain less defect zeolite membrane with high separation performances. Based on the results of this study, besides the utilization of additional chemicals such as organic solvent direction, modification to aging stirring and ball milling period are promising alternative methods. Future work will focus on systematic long-term testing and regeneration strategies to validate operational robustness under industrial conditions.

## Figures and Tables

**Table 1:** Summary FAU seed crystal and particle size characterization.

FAU Seed with Different Preparation Conditions		Crystallite Size (nm) by XRD	Micro Strain (%)	Relative Crystallinity (%)	Particle Size (nm) by SEM Images	DLS Analysis	
Stirring Time (h)	Ball Milling Time (h)					Particle Size (nm)	Polydispersity (nm)
4	-	36.7 ± 3.7	4.82	83.55	6106.99 ± 1075.92	431.95 ± 14.19	3.25 × 10^−6^
48	-	33.4 ± 4.7	5.40	95.53	985.35 ± 111.02	545.86 ± 35.30	1.90 × 10^−6^
144	-	24.6 ± 0.7	4.06	80.44	158.37 ± 33.42	450.30 ± 18.74	6.10 × 10^−7^
168	-	25.7 ± 1.2	3.94	83.9	125.04 ± 23.21	315.64 ± 10.02	5.85 × 10^−8^
168	6	27.6 ± 1.4	6.86	91.44	109.80 ± 4.04	372.77 ± 10.95	3.70
168	10	27.1 ± 0.5	8.28	79.72	95.51 ± 8.52	355.25 ± 1.96	5.72
168	12	26.1 ± 0.8	6.99	86.93	91.84 ± 14.63	297.22 ± 3.98	7.82 × 10^−7^
168	24	26.4 ± 2.5	6.03	87.77	72.08 ± 6.54	353.63 ± 2.00	13.00

## Data Availability

The original contributions presented in this study are included in the article/Supplementary Material. Further inquiries can be directed to the corresponding author.

## Supplementary Materials

The following supporting information can be downloaded at: https://www.mdpi.com/article/10.3390/membranes15120355/s1. Table S1: Comparison of PV separation performance of FAU (this work) with other membranes from literature. Refs. [6,45,47,48,49,50,51,52,53,54,55,56,57,58,59,60,61,62,63,64] are cited in Supplementary Materials.
