# Supplementary material for: Effect of Seed Size on Pervaporation Performances Through FAU Zeolite Membrane
Source: Membranes (Basel). 2025 Nov 25;15(12):355. doi: 10.3390/membranes15120355 (PMC12734560; doi:10.3390/membranes15120355)
Supplement: Supplementary file 1 [file membranes-15-00355-s001.zip › membranes-3986528-supplementary.pdf]

Supporting information

Effect of Seed Size on Pervaporation Performances Through FAU Zeolite Membrane

Alvin Rahmad Widyanto[1], Mikihiro Nomura[2,*]

[1] Regional Environment Systems Course, Graduate School of Engineering and Science, Shibaura Institute of Technology, 3-7-5 Toyosu, Koto, Tokyo 135-8548, Japan

[2] Materials and Chemistry Program, College of Engineering, Shibaura Institute of Technology, 3-7-5 Toyosu, Koto, Tokyo 135-8548, Japan

* Correspondence: lscathy@shibaura-it.ac.jp

The relative crystallinity of FAU zeolite based on following equation [1] with compared to the IZA standard database for FAU typical peaks.

$$\%RCFAU = SFC \cdot WF \cdot (SX/SR) \cdot 100\% \qquad \text{(Equation S1)}$$

where SFC is the scale correction factor given by SFx/SFR, which is the ratio of the scale factor of the zeolite samples to the scale factor of the standard sample (ZX). The SFC was adopted as 1 because all samples were measured under the same conditions. The WF is the peak width factor obtained by the ratio of the FWHM (Full Width at Half Maximum) of the peak (hkl = 533) of the respective zeolite samples by the standard sample (ZX). And the SX/SR ratio is the sum of the heights of the most intense diffraction peaks (around 6.23° (111), 10.11° (220), 11.86° (311) 15.58° (331), 18.58° (333), 20.25° (440), 22.66° (620), 23.485° (533), 26.89° (642), 29.42° (733), 30.55° (822), 31.20° (555), 32.24° (840), 32.98° (753), and 33.86° (664)) referring to each of the zeolite samples by the standard sample (ZX), respectively.

Table S1. Comparison of the water/IPA separation performance under similar test conditions for PV membranes.

Membrane	Temperature [°C]	Feed concentration [IPA/water wt%]	Total flux [kg m^{-2} h^{-1}]	Separation factor	Reference
FAU (seed; 4h aging)	75	80	0.56	3.20	This work
FAU (seed; 48h aging)	75	80	0.70	3.21	
FAU (seed; 144h aging)	75	80	0.50	21.69	
FAU (seed; 168h aging)	75	80	0.40	1759.39	
FAU (seed; 168h aging and 6h ball mill)	75	80	0.45	344681.34	
FAU (seed; 168h aging and 10h ball mill)	75	80	0.74	361038.05	
FAU (seed; 168h aging and 12h ball mill)	75	80	0.33	282.10	
FAU (seed; 168h aging and 24h ball mill)	75	80	0.39	44.96	
FAU	75	90	2.5	360	[2]
FAU	75	90	5.51	173	[3]
FAU	75	90	2.76	139	[3]
FAU	65	99.24	0.18	526	[4]
FAU (M1)	70	90	1.83	584	[5]
FAU (M2)	70	90	1.57	776	[5]
FAU (M3)	70	90	1.76	559	[5]
FAU (M4)	70	90	2.06	421	[5]
FAU (M5)	70	90	1.76	634	[5]
FAU (M6)	70	90	1.51	653	[5]
FAU (M7)	70	90	1.63	425	[5]
FAU (M7)	60	90	1.2	382	[5]
FAU (M7)	50	90	0.77	344	[5]

FAU (M7)	40	90	0.51	300	[5]
Zn-FAU	80	90	1.31	570	[6]
FAU (15 wt%) /PVA hybrid	40	90	0.059	1277	[7]
FAU (10 wt%) /PVA hybrid	40	90	0.051	891	[7]
FAU (5 wt%) /PVA hybrid	40	90	0.042	591	[7]
FAU (15 wt%) /PVA hybrid	50	90	0.073	809	[7]
FAU (10 wt%) /PVA hybrid	50	90	0.068	634	[7]
FAU (5 wt%) /PVA hybrid	50	90	0.056	400	[7]
Ag-FAU (5 wt%) /PVA hybrid	40	80	0.084	2717	[8]
NaY(10 wt%) /Chitosan	30	90	0.085	1850	[9]
NaY(15 wt%) /Chitosan	30	90	0.112	2400	[9]
NaY(20 wt%) /Chitosan	30	90	0.098	2100	[9]
NaA	75	90	4.88	40000	[10]
Mordenite	75	90	0.08	3974	[11]
Mordenite	75	90	0.658	4832	[11]
Mordenite	75	90	0.072	3702	[11]
Mordenite	75	90	0.039	3910	[11]
Mordenite	75	90	0.054	4312	[11]
Mordenite	75	90	0.1	3360	[12]
Mordenite	75	90	0.2	192	[12]
Mordenite	75	80	0.1	95.3	[13]
Mordenite, hollow fiber	75	90	1.45	6963	[14]
Mordenite, hollow fiber (seed size 4 μm)	75	90	4.35	5	[14]
Mordenite, hollow fiber (seed size 1 μm)	75	90	1.05	605	[14]
Mordenite, hollow fiber (seed size 0.3 μm)	75	90	1.40	4370	[14]
ZSM-5	75	90	1.4	1053	[15]
ZSM-5	80	95	0.14	501	[15]
CHA (SSZ-13)	105, VP	90	2.5	1500	[16]
CHA	75	90	10	82200	[17]
Choline chloride templated CHA	75	90	4.7	2000	[18]
Silica	80	90	0.65	73	[19]
Silica (Si/Zr)	80	90	0.86	300	[19]
Silica (Si/Zr)	80	90	0.67	27	[19]
Silica (Si/Ti)	80	90	0.78	400	[19]
Silica (Si/Al)	80	90	0.08	210	[19]
Silica (Si/(Al, Mg))	80	90	0.31	90	[19]
BTESE/PI	60	90	0.6	1300	[20]

References

1. Sousa, P.B.F.; Bieseki, L.; Pergher, S.B.C. Seed-Assisted Crystallization in the Hydrothermal Synthesis of FAU Zeolite from Acid-Treated Residue Glass Powder. *Materials (Basel).* **2025**, *18*, 1393. https://doi.org/10.3390/ma18071393.
2. Zhang, F.; Xu, L.; Hu, N.; Bu, N.; Zhou, R.; Chen, X. Preparation of NaY Zeolite Membranes in Fluoride Media and Their Application in Dehydration of Bio-Alcohols. *Sep. Purif. Technol.* **2014**, *129*, 9–17. https://doi.org/10.1016/j.seppur.2014.03.018.
3. Wang, Z.; Kumakiri, I.; Tanaka, K.; Chen, X.; Kita, H. NaY Zeolite Membranes with High Performance Prepared by a Variable-Temperature Synthesis. *Microporous Mesoporous Mater.* **2013**, *182*, 250–258. https://doi.org/10.1016/j.micromeso.2013.05.002.
4. Zhu, G.; Li, Y.; Zhou, H.; Liu, J.; Yang, W. FAU-Type Zeolite Membranes Synthesized by Microwave Assisted in Situ Crystallization. *Mater. Lett.* **2008**, *62*, 4357–4359. https://doi.org/10.1016/j.matlet.2008.07.026.
5. Wang, Q.; Guo, Y.; Xu, N.; Liu, Q.; Wang, B.; Fan, L.; Zhang, L.; Zhou, R. FAU Zeolite Membranes Synthesized Using Nanoseeds — Separation Mechanism and Optimization for the Pervaporation Dehydration of Various Organic Solvents. *J. Memb. Sci.* **2024**, *696*, 122522. https://doi.org/10.1016/j.memsci.2024.122522.
6. Zhu, M.; An, X.; Gui, T.; Wu, T.; Li, Y.; Chen, X. Effects of Ion-Exchange on the Pervaporation Performance and Microstructure of NaY Zeolite Membrane. *Chinese J. Chem. Eng.* **2023**, *59*, 176–181. https://doi.org/10.1016/j.cjche.2022.12.006.
7. Kulkarni, S.S.; Kittur, A.A.; Kariduraganavar, M.Y.; Davis, F.J. Pervaporation Dehydration of Isopropyl Alcohol with NaY Zeolite Incorporated Hybrid Membranes. *J. Appl. Polym. Sci.* **2008**, *109*, 2043–2053. https://doi.org/10.1002/app.28228.
8. Kwon, Y.; Chaudhari, S.; Kim, C.; Son, D.; Park, J.; Moon, M.; Shon, M.; Park, Y.; Nam, S. Ag-Exchanged NaY Zeolite Introduced Polyvinyl Alcohol/Polyacrylic Acid Mixed Matrix Membrane for Pervaporation Separation of Water/Isopropanol Mixture. *RSC Adv.* **2018**, *8*, 20669–20678. https://doi.org/10.1039/C8RA03474E.
9. Premakshi, H.G.; Ramesh, K.; Kariduraganavar, M.Y. Modification of Crosslinked Chitosan Membrane Using NaY Zeolite for Pervaporation Separation of Water–Isopropanol Mixtures. *Chem. Eng. Res. Des.* **2015**, *94*, 32–43. https://doi.org/10.1016/j.cherd.2014.11.014.
10. Hasegawa, Y.; Matsuura, W.; Abe, C.; Ikeda, A. Influence of Organic Solvent Species on Dehydration Behaviors of NaA-Type Zeolite Membrane. *Membranes (Basel).* **2021**, *11*, 347. https://doi.org/10.3390/membranes11050347.
11. Li, G.; Kikuchi, E.; Matsukata, M. Separation of Water–Acetic Acid Mixtures by Pervaporation Using a Thin Mordenite Membrane. *Sep. Purif. Technol.* **2003**, *32*, 199–206. https://doi.org/10.1016/S1383-5866(03)00035-2.
12. Lin, X.; Kikuchi, E.; Matsukata, M. Preparation of Mordenite Membranes on α-Alumina Tubular Supports for Pervaporation of Water–Isopropyl Alcohol Mixtures. *Chem. Commun.* **2000**, 957–958. https://doi.org/10.1039/A909867D.
13. Nakai, Y.; Widyanto, A.R.; Nomura, M. Water Permeation from IPA Solution Containing Sodium Chloride through MOR Zeolite Membranes. *J. Phys. Conf. Ser.* **2025**, *3107*, 12019. https://doi.org/10.1088/1742-6596/3107/1/012019.
14. Chen, C.; Cheng, Y.; Peng, L.; Zhang, C.; Wu, Z.; Gu, X.; Wang, X.; Murad, S. Fabrication and Stability Exploration of Hollow Fiber Mordenite Zeolite Membranes for Isopropanol/Water Mixture Separation. *Microporous Mesoporous Mater.* **2019**, *274*, 347–355. https://doi.org/10.1016/j.micromeso.2018.09.010.
15. Wang, Q.; Qian, C.; Guo, C.; Xu, N.; Liu, Q.; Wang, B.; Fan, L.; Hu, K. Pervaporation Dehydration Mechanism and Performance of High-Aluminum ZSM-5 Zeolite Membranes for Organic Solvents. *Int. J. Mol. Sci.* **2024**, *25*, 7723. https://doi.org/10.3390/ijms25147723.
16. Du, J.; Jiang, J.; Xue, Z.; Hu, Y.; Liu, B.; Zhou, R.; Xing, W. Template-Free Synthesis of High Dehydration Performance CHA Zeolite Membranes with Increased Si/Al Ratio Using SSZ-13 Seeds. *Membranes (Basel).* **2024**, *14*, 78. https://doi.org/10.3390/membranes14040078.
17. Hasegawa, Y.; Abe, C.; Ikeda, A. Pervaporative Dehydration of Organic Solvents Using High-Silica CHA-Type Zeolite Membrane. *Membranes (Basel).* **2021**, *11*, 229. https://doi.org/10.3390/membranes11030229.
18. Qiu, H.; Jiang, J.; Peng, L.; Liu, H.; Gu, X. Choline Chloride Templated CHA Zeolite Membranes for Solvents Dehydration with Improved Acid Stability. *Microporous Mesoporous Mater.* **2019**, *284*, 170–176. https://doi.org/10.1016/j.micromeso.2019.04.011.

19. Sekulić, J.; Luiten, M.W.J.; ten Elshof, J.E.; Benes, N.E.; Keizer, K. Microporous Silica and Doped Silica Membrane for Alcohol Dehydration by Pervaporation. *Desalination* **2002**, *148*, 19–23. https://doi.org/10.1016/S0011-9164(02)00647-1.
20. Liao, M.; Guan, H.; Zuo, H.; Ren, G.; Gong, G. High-Performance Flexible Hybrid Silica Membranes with an Ultrasonic Atomization-Assisted Spray-Coated Active Layer on Polymer for Isopropanol Dehydration. *Membranes (Basel).* **2024**, *14*, 154. https://doi.org/10.3390/membranes14070154.

Disclaimer/Publisher's Note: The statements, opinions and data contained in all publications are solely those of the individual author(s) and contributor(s) and not of MDPI and/or the editor(s). MDPI and/or the editor(s) disclaim responsibility for any injury to people or property resulting from any ideas, methods, instructions or products referred to in the content.
